# Nutrimetry and Evaluation of Intestinal Parasites and Anaemia in Malnourished Schoolchildren from Toliara (Madagascar)

**DOI:** 10.3390/children12020225

**Published:** 2025-02-13

**Authors:** Maria Valentina Alfano, Mónica Gozalbo, Gabriela Tapia-Veloz, Venny Guirao, Jose M. Soriano, María Trelis

**Affiliations:** 1 Department of Medicine and Public Health, Science of the Food, Toxicology and Legal Medicine, University of Valencia, 46010 Valencia, Spain; maval3@alumni.uv.es (M.V.A.); monica.gozalbo@uv.es (M.G.); 2 Area of Parasitology, Department of Pharmacy and Pharmaceutical Technology and Parasitology, University of Valencia, 46010 Valencia, Spain; gabriela.tapia@uv.es (G.T.-V.); maria.trelis@uv.es (M.T.); 3 Department of Health, ONG Bel Avenir, Toliara 601, Madagascar; agentdesante@ongbelavenir.org; 4 Food & Health Lab, Institute of Materials Science, University of Valencia, 46980 Paterna, Valencia, Spain; 5 Joint Research Unit on Endocrinology, Nutrition and Clinical Dietetics, University of Valencia-Health Research Institute La Fe, 46026 Valencia, Spain

**Keywords:** Madagascar, schoolchild population, stunting, thinness, nutrimetry, soil-transmitted helminth, protozoa

## Abstract

Background/Objectives: This study aimed to determine the malnutrition status, prevalence of intestinal parasites and anaemia, and the hygiene and sanitation conditions of children participating in the nutritional recovery programme at Las Salinas school of the ONG Bel Avenir in Toliara (Madagascar). The ultimate goal of the ONG Bel Avenir is to apply synergistic strategies to effectively combat malnutrition. Methods: A total of 49 schoolchildren aged 6 to 17 years enrolled in the nutritional recovery programme were studied. Data collection included sociodemographic information, hygiene and sanitation practices, and haematological and anthropometric measurements. Results: All participants were found to have anaemia. Regarding intestinal parasites, *Giardia intestinalis* was detected in 93.9% of cases, and *Trichuris trichiura* was identified in 28.6% of cases. Nutritional assessments revealed that 100% of the participants experienced thinness or acute malnutrition, while 32.6% exhibited stunting or impaired growth. Conclusions: The findings underscore the critical relationship between nutritional status and factors such as parasitology, haematology, and hygiene. Tools like the Nutrimetry assessment enable more specific diagnostics, guiding targeted interventions to address malnutrition. This study highlights the urgent need for policies and collaborative actions to improve the health conditions of the children in Madagascar.

## 1. Introduction

Madagascar is among the countries with the highest rates of malnutrition in the world. Malnutrition affects the entire population, with more impactful consequences on children [1,2]. Malnutrition is caused by various factors, including extreme weather conditions, food scarcity, a lack of clean water, and the poverty in which the population lives [3]. These factors contribute to weakening people’s health and to creating conditions for the development of infectious diseases [2]. The southern regions of Madagascar, with their extreme weather conditions such as long periods of drought and recurring cyclones and floods, are the most susceptible areas to food insecurity and its consequences [2].

According to data, 48.5% of Madagascar’s population was undernourished in 2021 [1]. Overall, the lack of food availability, market price fluctuations, and a low economic capacity to access food all limit the opportunity to have a diverse and quality diet. In 2020, 40.2% of children under 5 years old suffered from chronic malnutrition, a percentage that exceeds the thresholds established by the WHO and represents one of the highest in the world [1,2]. The main drivers of chronic malnutrition are poor diet, poor hygiene practices, a lack of access to clean water and other basic social services, a lack of knowledge and information, as well as unhealthy habits associated with cultural beliefs. It is also worth noting that less than 60% of children receive breastfeeding [2,3,4]. The area defined as the Greater South, during the peak of the scarcity season from November 2022 to March 2023, suffered a deterioration in relation to its food security situation [5]. The situation in southern Madagascar could worsen even further after Cyclone Freddy, which struck on 21 February 2023. According to projections, 115,000 children in these regions will need treatment for acute malnutrition this year. The most affected regions at the moment are the southern regions of Androy and southwestern Atsimo-Andrefana [6]. It has been observed that the manifestation of malnutrition in countries facing a scarcity of access to clean water, poor sanitation conditions, and poor hygiene practices—conditions similar to those in Madagascar—can be directly related to individuals harbouring parasites throughout childhood [7,8,9]. Nutritional deficiency can reduce the body’s immune function, thereby decreasing resistance to diseases and increasing susceptibility to intestinal parasites, including *Giardia intestinalis*, *Blastocystis* sp., *Ascaris lumbricoides*, and *Trichuris trichiura* [10,11,12]. These two determinants (low nutrient intake and the presence of infectious diseases) negatively influence the nutritional status of individuals. Some parasitic diseases fall under the category of Neglected Tropical Diseases (NTDs) [13]. These diseases act as barriers to development, especially among endemic populations, causing growth delays, affecting cognitive abilities, and being potential causes of disabilities that can be detrimental to an individual’s economic productivity [14,15]. In Madagascar, 70% of children aged 6 to 59 months and 49% of mothers have anaemia. Child anaemia rates are higher in Toliara (southwest region) at 76%, and lower in the Antananarivo region (north) at 64% [16]. Anaemia can be caused by various factors, including poor and/or inadequate nutrition leading to a lack of nutritional elements, the presence of parasitic infections, or hereditary disorders. The most common causes of anaemia are related to an iron deficiency, although a lack of vitamin B12, phosphate, and vitamin A have also been shown to cause anaemia [17]. The aim of this study is to evaluate the nutritional status, prevalence of intestinal parasites, and anaemia in malnourished schoolchildren participating in a nutritional recovery program in Toliara, Madagascar. By integrating anthropometric, haematological, and parasitological data, this study seeks to uncover the complex interplay between malnutrition, parasites, and anaemia and describe the epidemiological context of the school and where the children live.

## 2. Materials and Methods

### 2.1. Sample and Sampling

**Figure 1:**
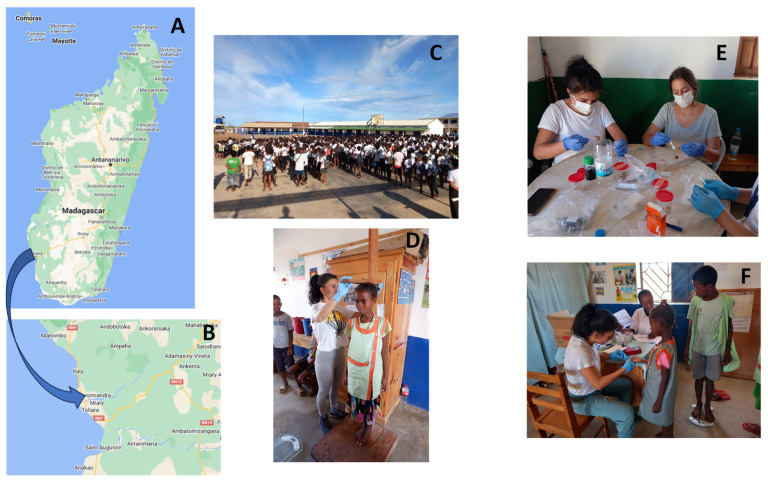
Location map of this study, situated in the southwest region of Madagascar (**A**) in the city of Toliara (**B**) in the Atsimo-Andrefana region, (generated with the ZeeMaps™ Programme (Zee Source, Cupertino, CA, USA)). This study took place at Las Salinas School (**C**), and anthropometrical assessments (**D**), samples of faeces (**E**), and blood (**F**) were collected and analysed.

This study consisted of an observational cross-sectional survey involving 49 schoolchildren (37% female and 63% male) in the nutritional recovery programme of Las Salinas school, affiliates to the ONG Bel Avenir in Toliara, the capital of the Atsimo-Andrefana region in Madagascar. The project was approved by the Research Ethics Committee in Humans (protocol number H1655289183675) of the Malagasy-Spanish Observatory on Nutrition and Food Security in the Developing World, with headquarters in the city of Antananarivo (Madagascar). The participants were aged between 6 and 17 years old, and from the peri-urban area of Toliara. Samples were collected and analysed in the laboratory dispensary located in the school itself (Figure 1). The schoolchildren were divided into three age groups based on the WHO 2007 references [18], as automatically generated by the WHO AnthroPlus™ [19] version 1.0.4 software for individuals aged over 5 years. This program categorizes individuals into three age groups: 5–9 years, 10–14 years, and 15–18 years [18]. In this study, these age groups included 8 children (16.3%), 32 children (65.3%), and 9 children (18.4%), respectively.

Informed written consent was obtained from the legal guardians of each participant, for data collection purposes. Fieldwork and sample collection were conducted between January and April 2023. Some remaining molecular analyses were conducted in the laboratory of the Faculty of Pharmacy at the University of Valencia from May to July 2023. The nutritional status of the participants was determined through an anthropometric assessment based on the WHO indicators [20] and Nutrimetry [21,22]. The participants underwent an interview to gather additional clinical information, and a sample of faeces and blood was collected for a coproparasitological and haematological diagnosis, respectively. The inclusion criterion was to belong to the school’s nutritional recovery programme and to attend school continuously.

### 2.2. Anthropometric Analysis

Weights and heights were measured using anthropometric equipment, an Omron™ electronic scale (precision, 100 g), and a Seca216™ stadiometer (precision, 1 mm), following the recommendations provided by the WHO [23]. The obtained results were analysed using the Who Anthro Plus™ version 1.0.4 software from the WHO for ages over 5 years [19]. This software includes an anthropometric calculator that, when provided with each participant’s data—age, sex, weight, and height—provides Z-score (Z) data for the following values: Height for Age (HAZ), Weight for Age (WAZ), and Body Mass Index for age (Z-BMI). The Z-score is a statistical measure indicating the deviation, expressed as Standard Deviation (SD), from the median of the studied value concerning reference patterns.

For children aged 5–19 years, the interpretation of height-for-age and weight-for-age remains consistent with that for children aged 0–60 months [20]. However, BMI-for-age uses different recommended cut-offs for overweight and obesity compared to preschool children [18,24]. Specifically, for children aged 5–19 years, a BMI-for-age of +1 SD aligns with the adult BMI cut-off of 25 kg/m^2^ at 19 years, indicating overweight. Similarly, +2 SD corresponds to a BMI of 30 kg/m^2^ at 19 years, marking obesity. A BMI-for-age of +3 SD or higher is classified as severe obesity, equivalent to a BMI above 35 kg/m^2^. A child whose weight-for-age (WAZ) falls in the range >+3 to >+1 may have a growth concern. That said, this is better evaluated using BMI-for-age indicators, which provide a more accurate assessment of nutritional status [24]. For thinness and severe thinness, the cut-offs are −2 SD and −3 SD, respectively, ensuring an age-appropriate assessment of nutritional status [18] (Table 1).

Undernutrition in children aged 5–19 years manifests in various forms, each with different characteristics and health implications. One such form, stunting, is identified by a low height-for-age and results from prolonged or recurring undernutrition. Stunting is commonly linked to factors such as poverty, inadequate maternal health and nutrition, frequent illnesses, and inappropriate feeding and care during early childhood. This condition not only impairs physical growth but also hinders cognitive development, preventing children from achieving their full potential and contributing to long-term health challenges [25]. The second form, underweight in children aged 5–10 years is defined as weighing less than expected for a healthy, well-nourished child of the same age and sex This measure, while useful in identifying undernutrition, cannot distinguish whether a child is stunted, wasted, or both, as it reflects weight without accounting for height [25]. Beyond 10 years of age, weight-for-age becomes less informative due to the onset of pubertal growth, a phase characterized by rapid and variable increases in height and weight. During this period, weight-for-age may misclassify taller children as having excess weight. To better assess nutritional status in children aged 10–19 years, BMI-for-age is recommended, as it accounts for both weight and height, offering a more accurate indicator of thinness, overweight, and obesity [19,24]. For individuals aged 5 to 19 years old, a validated form of undernutrition is recognized as thinness. Thinness refers to a child or adolescent whose weight is disproportionately low relative to their height, as determined by the sex-specific BMI-for-age index [24]. Depending on the degree of severity, the different conditions of undernutrition (stunting, underweight, and thinness) are classified as either moderate or severe (Table 1).

For a more comprehensive diagnosis of nutritional status, Nutrimetry was used, an epidemiological tool that can provide specific information about the malnutrition situation in population groups in different geographical areas and also help determine the origin of malnutrition problems [21,22]. Nutrimetry involves crossmatching data such as stunting Height-for-Age (HAZ) with BMI-for-age (Z-BMI) on a 3 × 3 table based on WHO references [18], resulting in 9 codes representing different nutritional status diagnoses (Table 2).

### 2.3. Coproparasitological Analysis

Through the coproparasitological analysis of stool samples, it is possible to detect eggs, larvae, cysts, and the oocyst of parasites associated with intestinal infections. The samples were collected during school hours, for which disposable sterile containers were provided to all the participants, distributing one per person. Fresh samples were immediately examined during fieldwork using the Kato–Katz technique, as described in the Bench Aids published by the WHO [26], to determine the presence of helminths eggs and provide a quantitative assessment. The remaining stool sample was processed for concentration with the REAL MidiSystem double-filter with a conical bottom system. This involved doble filtration and concentration by centrifugation with Total-Fix, following the manufacturer’s instructions (Durviz S.L., Valencia, Spain). The resulting concentrate was divided into two aliquots: one designated for optical microscopy, which was also analysed in the field, and the other for molecular techniques, analysed in the laboratory of the University of Valencia. The latter aliquot was first preserved in 70% ethanol for transport and then frozen at −20 °C to ensure the proper preservation of the parasitic forms until the analysis. Optical microscopy stands out as the predominant method employed for the etiological diagnosis, mainly due to its cost-effectiveness and minimal equipment requirements. This makes it particularly advantageous for application in resource-constrained countries. However, it has limitations in terms of sensitivity and specificity. Differentiation of species/genotypes/subtypes is often challenging using this technique. The efficacy of optical microscopy is influenced by various factors, including time constraints and, critically, the competence and experience of personnel in accurately visualizing and identifying diverse parasitic structures [27].

As a complement to optical microscopy, a molecular analysis technique was used for the diagnosis of *G. intestinalis*, the quantitative real-time Polymerase Chain Reaction (qPCR). DNA was extracted from 200 μL of the concentrated sediment obtained with the QIAamp DNA Stool Mini Kit (QIAGEN^®^, Hilden, Germany) according to the manufacturer’s instructions. The samples were analysed by a LightMix Modular Assay for *Giardia* spp. (manufactured by TIBMOLBIOL, distributed by Roche Diagnostics, Mannheim, Germany), which provided the primers, and a FAM labelled hydrolysis probe. For a final volume of 20 μL, each reaction mix contained 0.5 μL of the specific LMix assay, 10 μL of 2× master mix (PerfeCTa qPCR ToughMix, Quanta Biosciences, Gaithersburg, MD, USA), 4.5 μL of DNase/RNase-free water, and 5 μL of DNA. Negative (ultrapure water) and positive controls provided by the manufacturers for each of the parasites tested were included in each assay [28]. The PCR was carried out on the StepOnePlus Real-Time PCR System (Applied Biosystems, Waltham, MA, USA) linked to the StepOne software version 2.3., following the protocol indicated by the manufacturer. For *G. intestinalis*, the target of PCR amplification was a 62 bp fragment of the 18S rRNA gene. According to the information provided by the manufacturer, this assay will detect human *G. intestinalis* and animal *G. microti* species, but may not detect *G. ardeae*, *G. muris*, and *G. psittaci*.

### 2.4. Blood Analysis

For a haemoglobin analysis, capillary blood samples were collected on test strips and assessed using the HemoCue™ Hb 201+ System (HemoCue AB, Ängelholm, Sweden), which provides real-time results. This method delivers accurate Hb concentration measurements, and replicate sampling was conducted to minimize the within-subject error [29]. Haemoglobin concentrations were classified based on the WHO criteria: non-anaemia (Hb ≥ 12.0 g/dL for children aged 5–11 years, Hb ≥ 12.0 g/dL for children aged 12–14 years, Hb ≥ 13.0 g/dL for males aged 15–19 years, and Hb ≥ 12.0 g/dL for females aged 15–19 years), mild anaemia (Hb 11.0–11.9 g/dL for children aged 5–11 years, Hb 11.0–11.9 g/dL for children aged 12–14 years, Hb 11.0–11.9 g/dL for females aged 15–19 years, and Hb 11.0–12.9 g/dL for males aged 15–19 years), moderate anaemia (Hb 8.0–10.9 g/dL for all groups), and severe anaemia (Hb < 8.0 g/dL for all groups) [30].

### 2.5. Statistical Analysis

A statistical analysis was conducted using the results from the anthropometric, coproparasitological, and haematological assessments. The aim was to evaluate the impact of intestinal parasitosis on both the nutritional status (as measured by Nutrimetry) and the anaemic condition of the participants. The data were analysed using the Epi Info 6.0 software, a statistical program that calculates frequencies as well as means, and identifies the relationships between variables through the Chi-square independence test. The analysis was performed with a 95% confidence interval, and a significance threshold of *p* > 0.05 was applied.

## 3. Results

### 3.1. Anthropometric Results

The variables studied in the anthropometric assessments were calculated in relation to the total population (n = 49), by gender and age ranges. The Body Mass Index (BMI) Z-score (Z-BMI) simultaneously assesses height, weight, and age values (Table 1). After analysing the data, it was found that 73.5% (36/49) of the population presented severe thinness and 26.5% (13/49) had moderate thinness. Among the female participants, there was a prevalence of severe thinness: 61.1% (11/18), which is nearly 20% lower than the prevalence among the males who suffered from severe thinness in 80% (25/31) of cases. Regarding age ranges, it was observed that among the eight children aged 5–9 years, 50% (4/8) had severe thinness, 37.5% (4/8) had moderate thinness. The second group of children aged 10–14 years was the largest with 32 participants, of whom 75% (24/32) suffered from severe thinness, and 25% (8/32) had moderate thinness. The third group of 15–19 years old consisted of nine children, among whom 89% (8/9) had severe thinness, and the remaining 11% (1/9) had moderate thinness.

Z-Height-for-Age (HAZ) is a parameter used to evaluate growth delays (Table 1). Among the schoolchildren studied, 10.2% (5/49) were classified as experiencing severe stunting, 22.4% (11/49) were moderately stunted, and 67.3% (33/49) were within the normal height range. Regarding gender, the smallest proportion of participants were in the severe stunting category, with 11% (2/18) female and 9.7% (3/31) male. In the moderate stunting category, 27.8% (5/18) were girls and 19.3% (6/31) were boys. It was observed that 61.1% (11/18) and 70.9% (22/31) of the boys were within the normal height range, representing the majority in each group. In terms of ages groups, among the children aged 5–9 years, 25% (2/8) were stunted, while 75% (6/8) were within the normal height range. In the 10–14-year-old group, 12.5% (4/32) were severely stunted, 25% (8/32) were moderately stunted, and 62.5% (20/32) were within the normal height range. For the 15–19-year-old group, 11% (1/9) were severely stunted, 11% (1/9) were moderately stunted, and 77.7% (7/9) were within the normal height range. Applying Nutrimetry revealed that 67.3% (33/49) of the participants fell into Nutricode 3: Normal Stature–Wasting/Thinness (Normal HAZ–Low Z-BMI) and 32.7% (16/49) of them fell into Nutricode 1: Short stature/Growth Delay–Wasting/Thinness (Low HAZ–Low Z-BMI). Table 3 stratifies the results by gender and age, with the highest prevalence found among the 10–14-year-old schoolchildren, where 21.9% (7/32) and 40.6% (13/32) of the girls and boys, respectively, fell into Nutricode 3, indicating a suitable height for this age range. This contrasts with the percentages of 37.5% (12/32) in Nutricode 1, indicating underweight and short stature, which affects normal physical and mental development.

### 3.2. Parasitological Results

The parasitic species diagnosed in the study population were predominantly protists, followed by helminths. Among the protists, the most frequent species was *G. intestinalis* with a parasitisation percentage of 93.9%, followed by *Blastocystis* sp. with 87.8%, and amoebas: *Entamoeba coli* (36.7%), *Endolimax nana* (28.6%), *Iodamoeba bütschlii* (8.2%), *Entamoeba hartmanni* (6.1%), and *Chilomastix mesnili* (4.1%). Regarding helminths, two main species of geohelminths were observed, the whipworm, *Trichuris trichiura*, was present in 28.6% of cases, and the roundworm, *Ascaris lumbricoides*, in 6.1%. Another less prevalent helminth was the intestinal cestode *Hymenolepis nana*, which was present in 4.0% of cases. In terms of gender, the female population was as equally parasitized as the male. Interestingly, the entire population was parasitized with at least one parasitic species, and the prevalence of multiparasitism was remarkably high, with 46 children (93.9%) having more than one type of parasite. Among the age groups, the 5–9 group had higher percentages of *G. intestinalis* (100%; 8/8) and *T. trichiura* (37.5%; 3/8) than the older age groups.

### 3.3. Haematological Results

Anaemia was present in the entire study population. Of the 49 participants, 20.4% (10/49) experienced severe anaemia, while 79.6% (39/49) had moderate anaemia. Among the females, the prevalence of moderate anaemia was 27.8% (5/18), compared to 16.1% (5/31) among the males. The distribution of severe anaemia across age groups was relatively consistent, with 25.0% (2/8) of children aged 5–9 years, 18.8% (6/32) of children aged 10–14 years, and 22.2% (2/9) of children aged 15–19 years showing severe anaemia. These findings suggest that anaemia is widespread among the population, with minimal variation across genders and age groups.

### 3.4. Epidemiological Results

To evaluate the environment in which Malagasy children find themselves, it was established that the most common symptoms included abdominal pain and distension, diarrhoea, and fever, which could be associated with parasitic infections [31]. The symptom observed in the highest percentage of cases was abdominal pain (95.6%), followed by abdominal distension (91.3%). Regarding stool consistency, 77% identified their stools as liquid and pasty, and 75% reported pale-coloured stools, indicating intestinal disruption with a tendency toward diarrhoea; this may indicate the presence of intestinal parasites such as *G. intestinalis* [27]. Approximately 57% of the children reported chronic fever, 52% had headaches, and 47% experienced hives, mainly on their hands and feet. Regarding the hygienic-sanitary habits of the schoolchildren, notable habits included 100% of the boys and girls living in the peri-urban area and playing in the street. About 81% did not wash their hands after using the toilet, and 77% did not wash their hands before eating. Approximately 78% used well water for washing, and 72.7% drank well water. About 39% had contact with animals, primarily with pigs, followed by chickens, cats, and dogs. Additionally, 37% did not use latrines and practiced open-air defecation. The relative risk (Odds Ratio) associated with the probability of parasitic infection could not be determined, as all participants presented intestinal parasitism.

No statistically significant differences were found in the association of the variables studied, possibly because all of the participants share a similar nutritional status, are parasitized, and present anaemia. It is important to note that correlation does not imply causation, and our study was not designed to establish causal relationships. Additionally, since there is no control group and the focus of our study is on evaluating the epidemiological context of Las Salinas School, it has not been possible to determine statistically significant associations or infer causality.

## 4. Discussion

A summary of the results obtained in our study is shown in Table 4. The prevalence of thinness (Z-BMI) in the studied population was 100%, significantly higher than the prevalence of stunting (HAZ), recorded at 32.6%. Nutrimetry allowed for a comprehensive interpretation of these two variables, revealing that 67.3% of the thin children had a low weight but a normal height, while 32.6% exhibited both a low weight and low height. This finding suggests that a considerable portion of the population may have experienced an event during younger ages that led to stunted growth. Thinness, as an indicator of malnutrition, is applicable to individuals aged 5–19 years, but it is rarely reported in Madagascar. This is likely due to the focus of most nutrition surveys on children under 5 years old and their mothers [32], especially as the first five years of life are critical for growth and development [33].

Global development agendas [34,35] are increasingly adopting a more inclusive approach to addressing malnutrition by expanding the target monitoring groups. This includes not only children aged 5–19 to assess overweight and obesity but also women aged 15–49 to monitor thinness and underweight alongside overweight and obesity. However, schoolchildren aged 5–14 are not adequately represented or prioritized in global nutrition monitoring initiatives. Consequently, school-aged children are often overlooked in nutrition surveys, even though their nutritional status plays a crucial role in their health, cognitive growth, academic performance, and potential economic contributions in the future [32,36].

Since the Z-BMI indicator for individuals aged 5–19 years was introduced only in 2007 [24], its late adoption and absence from global indicators like the Sustainable Development Goals (SDGs) [37] may have contributed to its underutilization in research and national nutrition programs [32]. Additionally, the lack of data on national and provincial prevalence complicates efforts to compare findings across regions [36].

In this study, the prevalence of stunting is 32.6%, a percentage similar to what has been found in schoolchildren (5–14 years old) in the rural areas of Madagascar, where the percentage of schoolchildren with stunting was 34.9% [32]. In the different regions of Madagascar, stunting represents the highest percentage among the anthropometric indices measured in children under 5 years old [38,39]. At the same time, there is a lack of studies related to stunting in those over 5 years old and very few references at the national and global levels [36].

In Madagascar, the most widespread neglected tropical disease is soil-transmitted helminthiasis [14]. In twelve rural villages surrounding the Ranomafana National Park (RNP) in the Ifanadiana region, questionnaires and stool samples were collected from 574 subjects from random households. Through the Kato–Katz technique, the samples were examined, yielding the following results: 92.5% of the analysed population was infected with helminths. A total of 41% were aged 5 to 14 years, and in this age group, *T. trichiura* (84.6%) was the most detected [40]. *G. intestinalis*, as well as *Blastocystis* sp., also showed a very wide distribution among the child population. In two poor areas of Antananarivo, 410 stool samples of children aged 2 to 5 years were analysed, confirming a parasitic infection in 96.3% of cases, with *G. intestinalis* being the most prevalent species (79.5%), followed by *T. trichiura* (68.0%). In this study, the presence of *G. intestinalis* is related to the consumption of water from contaminated sources [38]. In the city of Mahajanga, in northwest Madagascar, the analysis of samples from 746 children from 14 different districts indicated a high presence of *G. intestinalis*, which could be related to the consumption of unsafe water and socioeconomic status. This study also showed that higher maternal education was associated with a lower presence of parasites. In the neighbourhoods with poor hygiene and limited sanitation, there was a predominance of *G. intestinalis* and *E. coli* [41]. At Las Salinas school, it is routine to administer the Albendazole deworming treatment to the children twice a year as a measure to control geohelminthiasis in schoolchildren. As part of this practice, all the schoolchildren received their dose in December 2023, and this may have influenced the prevalence detected in this regard. Despite this effort, there has not been a significant impact observed among the group of undernourished children, probably because community health and environmental conditions have not been controlled at the same time.

The relationship between malnutrition and parasitic infections is very close and demonstrable. In the Malagasy rural areas of Moramanga and Morondava, the highest probability of stunting was related to the presence of *T. trichiura*, which can remain in the digestive tract for years, leading to a chronic infection that can affect growth [42]. It is worth noting that *A. lumbricoides*, *T. trichiura*, and *Ancylostoma* duodenale/*Necator americanus* are among the most widespread parasites globally and are responsible for the deaths of 135,000 people per year [41].

The presence of helminths can reduce the available nutrients, contributing to blood loss and causing anaemia [43]. In a study of the relationship between anaemia, soil-transmitted helminthiasis, malnutrition, and well-being, almost all the participants with parasitic infections also suffered from anaemia. People living in poverty and suffering from anaemia were more likely to harbour intestinal parasites [44]. The action of infectious agents in the human body can lead to problems in nutrient absorption, resulting in anaemia and other pathologies. At the same time, a circular relationship is created between intestinal parasitism, malnutrition, and anaemia, with all of these feeding into each other. An association of anaemia with the consumption of contaminated water, nutritional status, age, gender, poverty, rural living, and a deficient diet has been demonstrated, suggesting that more studies are needed on social determinants [45].

## 5. Conclusions

This research underscores the importance of expanding malnutrition assessment criteria beyond conventional measures and conducting studies that encompass older age demographics.

To address these challenges, the World Health Organization (WHO) recommends collecting anthropometric measurements for adolescents whenever possible, including during primary health services, to monitor both undernutrition and overweight [46]. Despite this recommendation, there remains limited evidence and experience regarding the most appropriate anthropometric measurements and methods for classifying nutritional status among adolescents. The interpretation of these measurements is further complicated by puberty and variations across ethnic groups [47,48]. Nevertheless, focusing on adolescent nutrition through effective anthropometric assessments could improve their health during adolescence, enhance their nutritional status in adulthood, and ultimately break the cycle of intergenerational malnutrition by improving the health of future generations.

The simultaneous presence of malnutrition and parasitic infections such as *G. intestinalis* and *T. trichiura* indicates the complex interplay between inadequate nutrition and infectious illnesses, resulting in detrimental health outcomes. Nutrimetry emerges as a valuable tool for gathering and analysing data by cross-referencing key anthropometric indicators like HAZ and Z-BMI, enabling a comprehensive interpretation at both the epidemiological and clinical levels. This methodology sheds light on the prevalence of different malnutrition forms suggesting varied underlying causes and vulnerabilities. Such an approach facilitates pinpointing individual malnutrition origins and tailoring the appropriate interventions.

Moreover, the analysis emphasizes that malnutrition and the associated factors such as parasitic infections pose significant public health challenges. Addressing these issues necessitates a collaborative, multisectoral approach and a strong political commitment to implement preventive measures that account for the diverse causes of malnutrition and proactively mitigate them.

## Figures and Tables

**Table 1:** Children growth indicators adapted from WHO Standards [19] and WHO Reference [18].

Z-Score	Growth Indicators 5–19 Years Old		
	Height/Age (HAZ)	Weight/Age (WAZ) Only for 5–10 Years	BMI/Age (Z-BMI)
≥+3 SD	Tall stature	See BMI-for-age	Severe obesity
<+3 SD to ≥+2 SD			Obesity
<+2 SD to ≥+1 SD	Normal		Overweight
<+1 SD to >−1 SD		Normal	Normal
≤−1 SD to >−2 SD		Slight thinness	Slight thinness
≤−2 SD to >−3 SD	Moderate stunting	Moderate underweight	Moderate thinness
≤−3 SD	Severe stunting	Severe underweight	Severe thinness

**Table 2:** Nutricodes, adapted from Selem-Solis et al. [23].

	BMI/Age		
Height/Age	Thinness Z ≤ −1 SD	Healthy Weight Z > −1 SD y < +1 SD	Overweight/Obese Z ≥ +1 SD
Tall stature ≥+2 SD	Nutricode 5 Tall stature- Wasting	Nutricode 6 Tall stature- Healthy weight	Nutricode 11 Tall stature- Overweight
Normal stature <+2 SD to >−2 SD	Nutricode 3 Normal height– Wasting/Thinness	Nutricode 4 Normal height– Healthy weight	Nutricode 9 Normal height– Overweight
Short stature ≤−2 SD	Nutricode 1 Short stature/Stunting– Wasting/Thinness	Nutricode 2 Short stature/Wasting– Normal weight	Nutricode 7 Short stature/Stunting– Overweight

**Table 3:** Frequencies (%) of the studied population by Nutrimetry, stratified by gender and age group. (n_f_: Number of female participants in this study; n_m_: number of male participants in this study; and CI: confidence interval, a statistical measure indicating the reliability of an estimate).

BMI	z ≤ −1		z ≤ −1		z ≤ −1	
	5–9 Years n = 8		10–14 Years n = 32		15–19 Years n = 9	
**HAZ**	n_f;%_ (CI (95%))	n_m;%_ (CI (95%))	n_f;%_ (CI (95%))	n_m;%_ (CI (95%))	n_f;%_ (CI (95%))	n_m;%_ (CI (95%))
Z < +2 or >−2	2; 25 (4.5–64.4)	4; 50 (17.5–82.6)	7; 21.9 (9.9–40.4)	13; 40.6 (24.2–59.2)	2; 22.2 (4.0–59.8)	5; 55.6 (22.7–84.7)
z ≤ −2	2; 25 (4.5–64.4)	0	5; 15.6 (5.9–33.6)	7; 21.9 (9.9–40.4)	0	2; 22.2 (4.0–59.8)

**Table 4:** Summary of the results obtained in the studied population from the peri-urban area of Toliara.

Analysed Parameter	5–9 Years (61–120 Months)		10–14 Years (121–180 Months)		15–19 Years (181–228 Months)	
	♀	♂	♀	♂	♀	♂
*Nutritional status (Z-BMI)*						
Slight thinness ≤−1 SD to >−2 SD	1/8 (12.5%)	0	0	0	0	0
Moderate thinness ≤−2 SD to >−3 SD	1/8 (12.5%)	2/8 (25%)	4/32 (12.5%)	4/32 (12.5%)	1/9 (11.1%)	0
Severe thinness ≤−3 SD	2/8 (25%)	2/8 (25%)	8/32 (25%)	16/32 (50%)	1/9 (11.1%)	7/9 (77.7%)
*Nutritional status (HAZ)*						
Normal <+2 SD to >−2 SD	2/8 (50%)	4/8 (50%)	7/32 (21.9%)	13/32 (40.6%)	2/9 (22.2%)	5/9 (55.6%)
Moderate stunting ≤−2 SD to >−3 SD	2/8(50%)	0	3/32 (9.4%)	5/32 (15.6%)	0	1/9 (11.1%)
Severe stunting ≤−3 SD	0	0	2/32 (6.3%)	2/32 (6.3%)	0	1/9 (11.1%)
*Nutritional status (Nutrimetry)*						
Nutricode 1 (Short stature/Stunting– Wasting/Thinness)	2/8 (25%)	0	5/32 (15.6%)	7/32 (21.9%)	0	2/9 (22.2%)
Nutricode 3 (Normal height–Wasting/Thinness)	2/8 (25%)	4/8 (50%)	7/32 (21.9%)	13/32 (40.6%)	2/9 (22.2%)	5/9 (55.6%)
*Prevalence of parasites*						
*Giardia intestinalis*	4/8 (50%)	4/8 (50%)	12/32 (38.4%)	19/32 (59.4%)	1/9 (11.1%)	7/9 (77.7%)
*Blastocystis* sp.	4/8 (50%)	4/8 (50%)	10/32 (31.3%)	16/32 (50%)	2/9 (22.2%)	7/9 (77.7%)
*Entamoeba coli*	0	1/8 (12.5%)	6/32 (18.8%)	6/32 (18.8%)	1/9 (11.1%)	4/9 (44.4%)
*Endolimax nana*	0	2/8 (25%)	6/32 (18.8%)	4/32 (12.5%)	0	2/9 (22.2%)
*Iodamoeba bütschlii*	0	0	2/32 (6.3%)	2/32 (6.3%)	0	0
*Entamoeba hartmanni*	0	0	2/32 (6.3%)	1/32 (3.1%)	0	0
*Chilomastix mesnili*	0	0	2/32 (6.3%)	0	0	0
*Trichuris trichiura*	1/8 (12.5%)	2/8 (25%)	5/32 (15.6%)	3/32 (9.4%)	0	3/9 (33.3%)
*Ascaris lumbricoides*	0	0	1/32 (3.1%)	1/32 (3.1%)	0	1/9 (11.1%)
*Hymenolepis nana*	0	0	1/32 (3.1%)	0	0	0
*Prevalence of anaemia*						
Moderate anaemia	2/8 (25%)	4/8 (50%)	10/32 (31.3%)	17/32 (53.1%)	1/9 (11.1%)	5/9 (55.6%)
Severe anaemia	2/8 (25%)	0	2/32 (6.3%)	4/32 (12.5%)	1/9 (11.1%)	1/9 (11.1%)

## Data Availability

The data presented in this study are available on request from the corresponding author.
